# Perspective: Limiting Antimicrobial Resistance with Artificial Intelligence/Machine Learning

**DOI:** 10.34133/bmef.0033

**Published:** 2023-12-15

**Authors:** Daniel Amsterdam

**Affiliations:** Jacobs School of Medicine & Biomedical Sciences, State University of New York, Buffalo, NY, USA.; Departments of Microbiology and Immunology, Medicine, and Pathology at the Jacobs School of Medicine and Biomedical Sciences, University at Buffalo, Buffalo, NY, USA.; Department of Laboratory Medicine, ECMC, Buffalo, NY, USA. Address correspondence to: amsterda@buffalo.edu

## Abstract

The author traces his experience with the application of computers in clinical microbiology over the past 60 years, specifically in directing clinicians to treat bacterial infections diagnosed by the laboratory and the antibacterial agent(s) that could be used to treat those infections. Appropriate use of antibiotics will result in reduced antimicrobial resistance, which is increasing worldwide. An early form of AI, Mycin (1976), a system based on rules provided by experts designed to propose antibiotic regimens for central nervous system infections, was never applied due to the limitations in the number of rules that could be incorporated into the clinical workflow. Machine learning (ML) was developed to overcome the limitations of expert systems. Several variables that influence the outcome bacteria/drug interaction, such as the source of the infection, absence of antimicrobial resistance markers, patients’ health profile, and the historical susceptibility within the hospital and the local area are incorporated in the proposed comprehensive AI/ML program. The role of AI in the discovery of new antimicrobial agents is also addressed.

For almost 60 years, I have been concerned and engaged with the accumulation, counting, sorting, and stratifying of clinical microbiology test results and data with computer-assisted devices [1–4]. Considering artificial intelligence (AI) and its associated component, machine learning (ML)’s omnipresence in every aspect of our lives (ethical, cultural, political, and economic), it would seem necessary that it should now be consistently incorporated in medical discovery and decision support.

My first experience with an elementary form of AI in healthcare was with expert systems, such as Mycin. Systems are designed and based on rules provided by experts that could then be translated and programmed. Mycin, the initial expert system developed in 1976, was directed to propose antibiotic regimens for central nervous system infections [5]. It was never actually used because of the limitation, given the number of rules and obstacles to incorporate the information into the clinical workflow. It should be noted that ML was developed to overcome the limitations of expert systems. ML can be considered as a machine-associated reasoning or diagnostic tool.

In general, clinical laboratories have and follow procedures to ensure compliance and accurate and reliable test results. In this way, quality is maximized, and liability risk is minimized. A high-quality clinical laboratory process consists of 3 phases: preanalytical, analytical, and postanalytical. It is within this last phase that results are provided to the clinician upon which to act or continue to observe the status of the patient. For a clinical microbiology laboratory, the test request for a “C&S” (culture and susceptibility) requires that for each microbial species isolated, susceptibility or resistant end points are usually reported for 12 antibiotics. The interpretive category: susceptible (S), intermediate (I), or resistant (R) incorporates clinical information as to the known (Food and Drug Administration package insert) achievable level of drug/antibiotic in the body site (e.g., blood, urine, and spinal fluid). The S/I/R results do not include other clinically relevant information as to the nature of infection—community acquired or hospital acquired (nosocomial), patient allergenicity, genetic resistant marker(s) of the microorganism, and historical performance of the drug for the microorganism (bug/drug combo) in the geographic area of the institution or the institution itself (antibiogram). Other factors can influence the clinical course and outcome of microbial disease such as the hematologic profile (neutrophil function), weight, nutritional and health status, and thyroid function of the patient.

The risk of antimicrobial resistance was considered nearly 80 years ago by A. Fleming. When he was awarded the Nobel Prize for his discovery of the inhibitory effect of *Penicillium notatum* on the growth of several genera of Gram-positive bacteria, namely, *Staphylococcus*, *Streptococcus*, *Pneumococcus*, and the gonococcus, he noted in his Nobel prize acceptance speech, “It is not difficult to make microbes resistant to penicillin in the laboratory, and the same has happened in the body”. It turned out that soon after the introduction of penicillin into the pharmaceutical armamentarium, it became of limited use. Today, we recognize that antimicrobial resistance is the outcome of antibiotic usage.

In a recent World Health Organization report, high levels of resistance in bacteria causing life-threatening bacteremia and other infections were well documented on the basis of data reported by 87 countries in 2020 [6]. The Global Antimicrobial Resistance and Use Surveillance system (GLASS) report analyzes antimicrobial resistance rates from 127 countries since 2017. In addition, it reports data on antimicrobial use in 27 countries. The report cites resistance levels above 50% for bacteria that cause bloodstream infections, such as *Klebsiella pneumoniae* and *Acinetobacter* spp. These 2 bacterial species are included in the ESKAPE group, an acronym comprising *Enterococcus faecium*, *Staphylococcus aureus*, *K. pneumoniae*, *Acinetobacter pneumoniae*, *Pseudomonas aeruginosa,* and *Enterobacter* spp. GLASS reported that 8% [7] of bloodstream infections caused by *K. pneumoniae* were reported as resistant to the carbapenems, considered “last resort” antibiotics. Sixty percent of the frequently associated agent of sexually transmitted infections, *Neisseria gonorrhoeae,* were resistant to oral ciprofloxacin. An additional antimicrobial resistance (20%) was reported for *Escherichia coli*, the common agent of urinary tract infections, for first-line drugs, ampicillin and trimoxazole, and second-line fluoroquinolones.

For these clinical and laboratory needs, some time after the introduction of Mycin, I had considered that an evidence-based clinical decision support should be incorporated in the postanalytical phase of the “C&S” test/report. For many years, there have been reference materials, including Antibiotics in Laboratory Medicine [8], and 2 clinical resource tools available to “expeditiously” address decision-making. Up To Date and the Cochrane Reviews are published resources that provide data and use evidence-based clinical decision information that is screened with the aim of minimizing bias. The “editor” for appending such an informed report is a regional or national expert; that objective was never achieved. Considering that clinically significant microorganisms continually develop new resistance patterns, reporting timely datasets that detail resistance will always be challenged by the need to evaluate “yesterday’s” susceptibility data. We must recognize resistance in real time to move forward. Clearly, AI/ML support would expedite and advance this process.

In their overarching review on the attributes of AI/ML for gaining advantages against infectious diseases, Wong et al. [9] have limited their discussion of the application in everyday needs of clinicians treating patients with infections in the clinic or hospital. What I am considering here is that an AI/ML tool should be implemented, either by an organization, healthcare system, or medical specialty to assist medical practitioners to administer the “right” antimicrobial agent, at the “right” time for the “right” time duration for the “right” patient, representing the Institute of Medicine tenets for optimal healthcare [10]). The Institute of Medicine guidelines do not preclude the clinician from risk due to their selection of therapies. When using AI/ML as an adjunct to guide treatment, I would suggest that a statement such as “the treatment decision for this patient has been augmented with AI/ML support” should be included in electronic health record documentation. Ultimately, the treating clinician makes the decision to administer the antimicrobial agent(s) from a liability perspective.

The variables included in such a comprehensive AI/ML program would include the following:

• Antimicrobial agent with the greatest probability of efficacy, lowest toxicity, and shortest duration of treatment.
• Adherence to local susceptibility data:
  º Program should be adjustable for a distinct geographic location.
  º AI/ML technology, such as Google Vertex, should be utilized by the local hospital’s information technology professionals to incorporate the local antibiogram data into the program.
• Absence of antimicrobial agent resistance marker(s).
• Delivery of the correct dose according to site of infection (such as central nervous system, bone, blood stream, and/or urinary tract) and patient’s clinical presentation.
• Patient type and combination of comorbidities, such as diabetes, immunosuppression, obesity, older age, and nutritional status.
• Capability and timing of changing route of administration from parenteral to oral.
• Other factors as deemed necessary (case by case).

AI has also been proposed and is being explored to discover new antibiotics. Given that antibiotic development is slow, failure-prone, and expensive, a limited number of new medicines have been developed in the past decade. Therefore, it would be prudent to explore alternative approaches. AI affords that opportunity in the discovery of small molecule antibiotics, antimicrobial peptides, and computer enhanced drug design to address this challenge [9,11].

We look forward to witnessing AI discovery of new drugs and directing the design of drugs that are bactericidal, possessing a greater spectrum of activity with diminished potential for resistance. Moreover, determining the antimicrobial armamentaria in hospital/health system formularies and clinics using AI/ML technology is a forward-thinking approach to address resistance concerns now and in the future.
